# A *Fondrakö* text: Cultural religious tradition and social integration of community

**DOI:** 10.12688/f1000research.127772.1

**Published:** 2023-01-11

**Authors:** Sonny E. Zaluchu

**Affiliations:** 1 Teologi, Sekolah Tinggi Teologi Baptis Indonesia, Semarang, Jawa Tengah, 50141, Indonesia; 2 Sosiologi Agama, Universitas Kristen Satya Wacana, Salatiga, Jawa Tengah, 50711, Indonesia

**Keywords:** local wisdom, fondrakö, sociology, social binding, Durkheim, community

## Abstract

Fondrakö is the local wisdom of the Nias ethnic community and has two functions. First are the oral religious holy book teaching ancestors' values and philosophy. Second is the highest consensus institution of the traditional society formulating and making decisions on the socio-religious law to guarantee the order of the community. This paper explains that social differentiation has happened in the modern era; philosophical values of *fondrakö* remain to live up to this day and experience the proliferation of rites. The argumentation built through this paper is that primitive culture will not simply disappear in the modern community just because it experiences accommodation and the decrease of emotional intensity into new forms. Social laws of primitive religion are not always contradictory and can run parallel with civilization.

## Introduction

The ethnic religious texts, in the beginning, did not have the early written form. The contemporary final form is the transformation from the traditional format of oral products or oral law collection. The research results of Tsuria, Bellar, Campbell, and Cho (2021) conclude that, along with time, holy books and religious texts experienced the shift from oral composition uttered in religious meetings and rites to become written texts in a form of copies on animal skin or papyrus paper and last to be written in a book format after the revolutionary invention of a printing device. In other words, the ancient religious texts were built in the oral tradition ( Clark, 2005) and then developed to become written documents as long as the advanced printing technology ( Long & Long, 2017). Although text transmission change has happened, Widder (2017, p. 2) emphasized that the power of those texts relies on the linguistic aspect more than its form. According to Widder (2017), language becomes the best media in delivering religious messages by listening to what is talked about or delivered orally. Therefore, in various traditions, the transmission of religious messages and teachings has started in a form of the oral method. Especially before the writing and printing technology took over, oral tradition was rooted deeply in the way the community built their spirituality. Through language, communities define their belief system and build symbols to communicate in the system horizontally.

*Fondrakö* is one of the oral traditions developed in the ancient Nias ethnic community but is still influential in the contemporary community life. The values and teachings in this tradition still remain as local wisdom in the social life order of the Nias community, even though the majority of Nias people have converted into Christian followers and have abandoned their ethnic religion ( Harefa, 2013; Mendrofa, 1981). The reality is the laws of *fondrakö* are in line with the teachings of Christianity and is even feared and obeyed more than religious instructions. This is a crucial matter because the values of *fondrakö* rely on ethnic religion and customs, not on the teachings of Christian religion sourced from Divine revelations. *Fondrakö* is the cultural product from a primitive community, which at the beginning took root in myths, traditions, rituals, natural power, and is pagan in the animism-dynamism perspective. Because the community tend to give more attention to traditional values than to the teachings of the holy book, on the one hand, the clashes of values and behavior often happen, and there will be the combination of teachings or there appears syncretism, on the other. This occurs because in the perspective of the local inhabitants, *fondrakö* is not merely the values arranging social relationship and kinship but also inclusive as spiritual teachings from an oral ‘religious text’. The relationship of both is inseparable. Since Nias people live and actualize as a community, *fondrakö* becomes the referent values side by side with the spiritual aspect.

This paper aims to comprehend deeply why *fondrakö* becomes a discourse power that keeps surviving in the modern era. Exploration toward values and power of *fondrakö* is expected to provide contribution for the anthropological studies of culture and religious sociology, which will complete the literature on unification of culture and religion. For that, the first thing to do is to introduce the origin of *fondrakö* with two approaches, myths and sociology. Then the discussion will dig out the power of *fondrakö* in its tradition root as it became a covenant, social binding, and its integration in a belief system or ethnic religious spirituality. There are not many that have conducted specific exploration on the law of *fondrakö* and analyzed it in the concept of sociology. Several research results reported are more about the practical implementation of *fondrakö* in various fields of life and do not touch the socio-religious approach as discussed in this paper. The research of Harefa (2013), for instance, only descriptively explained the concept of *fondrakö* as a customary product. The power of *fondrakö* is seen as a tradition producing local wisdom. Therefore, the research of Zega (2019) elaborated the implementation of the cooperation workshop as arranged in *fondrakö* and in the modern leadership management. Markus (2004), although not specifically reviewing *fondrakö*, in his thesis on intercultural mission related to arts and handicraft mentioned that *fondrakö* is related to the initiation of a clan leader in Nias. The quite complete research was conducted by Gulo (2011) writing a thesis on the relationship between *fondrakö* and the concept of *böwö*, namely customary dowry in the Nias wedding scheme. The research on *fondrakö* as social binding and spiritual construction as the gap that will be addressed in this paper. The temporary belief is that *fondrakö* is not only a customary entity but also part of a belief system that moves various strata of the community social life in a consensus.

## Methods

### Ethical considerations

This article followed all ethical standards for research without direct contact with human or animal subjects.

This paper will be arranged in four steps to achieve the research objectives. First, theoretically explaining sacred texts and books in a cultural context. Then explaining the relationship between *fondrakö* and Nias people's religiosity. A more specific discussion in the third part concerns the blessing and curse of *fondrakö* as moral and supernatural force that builds people’s fear of not violating it. The last section is an analysis of how *fondrakö* plays a role in the formation of social binding and modernity challenges. Discussions rely on primary data from the literature on Nias in the form of books and research results published in journals. Web searches and library studies were conducted to collect these books. All of these sources have become references cited in the article.

Thus all of this research is qualitative Marying (2004), which is carried out with a literature study to construct a theory about *fondrakö.* There are still very few references to *fondrakö* and Nias culture, so all books and articles researched on Nias topics must be used. Several parts of that research provide data and notes about *fondrakö.* The research began with initial observations in a traditional Nias village. The researcher unilaterally decided on a traditional village chosen as the object of observation and observed social behavior in joint decision-making based on *fondrakö* values. Observations were also focused on seeking answers as to whether *fondrakö* was still valid and to what extent it had influenced community decision-making. The observation results become a phenomenon analyzed and constructed using the sociology of religion approach. This approach was chosen because the observation group is a Christian community. However, they still practice *fondrakö* traditional values even though they are aware that the values of the two have mixed in the people's perspective. Decisions taken within the group are universal and binding and affect the social dynamics of society. The sociology of religion approach was chosen because *fondrakö* directly impacts social interaction in society and is part of a belief system. As is known, sociology looks at religion, not from the point of view of human relations with God (theology). In contrast, sociology sees religion as about society and its impact on society. From this perspective, the sociology of religion will explain how religion, both primitive religion through *fondrakö* and mainstream religion, collaborate in shaping culture, behavior, and social interactions. *Fondrakö* is part of the culture of the inclusive Nias people, which is directly related to religious life.

## Results

### Theory of religious holy books

Every religion has its own religious holy book. Through the religious holy book, the followers have doctrinal guidance on the principles of belief, values, and statements on what they believe in, the teachings that are believed in and considered holy, the wisdom to live well both individually and in community, and the guidance to approach or understand the transcendent God ( Wilson, 1991). Basically, the religious holy books are not far different from the texts born in a form of oral literary works which are then written in a book. What makes them different, according to Madigan (2013), is the attitude and treatment toward the text. Madigan (2013) stated that when a community of the believers considers and admits a text has sacredness, dignity, and can be accepted as a truth to strengthen faith and religious life, the text by itself is categorized as a religious holy book. If it has been accepted as holy writing, the influence of the text spreads, not only reaching the religious area but also covering the political, social, and economic life of the community. Furthermore, Madigan explained that the texts in a holy book are important elements in a complex process of religious follower identity formation ( Madigan, 2013). Through the texts in a holy book, religious followers can hear God in the middle of the community ( Siddiqee, 2013). In other words, the religious holy book texts, are on one hand informative, and on the other, performative because they are related directly in the religious follower community identity formation. The relation of both forms the locus of belief in the middle of the community. Therefore, the religious holy books always correlate with the way religious followers express their social life ( Ritzer & Stepnisky, 2019a, p. 111).

At the beginning, religious holy books did not appear in a contemporary form. The information contained inside them as the content of a holy book took the more primitive form when first introduced in society, namely utterances and oral communication. Durkheim stated that analyzing the religious contemporary form only manages to be done when that religion is learned in its most primitive form ( McKinnon, 2014). In the religious holy book context, the thinking of Durkheim can be accepted because the truth is the religious holy books that we receive today are the transformation results from their primitive form sourced from animism, naturism, and totemism ( Durkheim, 2011). In that primitive form, the religious holy books did not have their current form. The informative and performative form of religious holy books is stated in symbols, abstraction, rituals, and other oral forms. As time went by, the texts experienced shifts and form change. In the beginning they were delivered in the oral and verbal composition, then they were written and stated in a form of reading, and then transferred in various forms of manuscript copies and finally arrived in a contemporary form in a book or digital application ( Tsuria *et al.*, 2021).

By taking the examples of the dichotomy of holy-forbidden, holy-unholy from the context of The Old Testament, Hundley explained that religious texts in general play a role in providing clues to the followers on sacrality ( Hundley, 2013). Sacred space formed from religious texts become direction and guidance in living a pious life and on how to behave religiously in a secular world. This is inseparable from the way the group of community interpret religious texts and apply them in their life. According to Sanders, the hermeneutic pattern always relies on two points. The first reference is a text as tradition that then becomes the behavior pattern in a context and situation ( Sanders, 1987, p. 173). In this case, it is seen that religious behavior and social behavior are also influenced by how far a group of community interprets their religious texts. In regard to this, Shillington ( Shillington, 2002, p. 63) revealed that religious texts are from the unity of language, texts, and oral utterances that encourage intention inside the religious followers to behave according to the content of their holy book. Therefore, religious holy books can be seen as a rule that internalizes and binds the religious followers to carry out their role in the social order. Berger & Luckmann (2018, p. 21) supported this conclusion through his theory on social construction. Religious holy book texts can become an individual knowledge potential that can be developed to form reality in the middle of the community. We can conclude from that statement that social behavior basically runs parallel with the community religiosity aspect itself. Both exist in the dialectical relationship. Practically, that social behavior is the reflection of interpretation and internalization of religious values through religious holy books.

### *Fondrakö* and religiosity

Validation of *fondrakö* was conducted in a rite led by *ere.* In the ancient Nias belief, *ere* was the religious *imam* ( Laiya, 1983, p. 24). The meaning of *ere* as the local religion *imam* was also introduced by the missionaries that came to Nias when they spread the Christian religion ( Hummel & Telaumbanua, 2007; Sundermann, 1905; Zaluchu, 2021). The original meaning of *ere* is an expert or specialist in a certain field. Therefore, the function of *ere* was often associated with religious rites and rituals, so the position was made to be specific in the field of religion.

The activity center of *ere* was a meeting house named *osali.* In *osali*, *ere* led the ceremonies and rituals in front of the inhabitants and provided explanation and interpretation on the laws of the custom and religion. *Osali* did not only become a public space but also functioned as a religious space. This idea was then adopted by the Western missionaries when they changed the function of *osali* to become the worship place for Christians. Therefore, *ere* had the key role in the rite management for the need of the custom and religion of the community. Its position was equal with a ruler and was very respected. *Ere* was even considered as a holy person because he had the supernatural ability, namely as a mediator to enter the spirit world. The *imam* was believed as the traffic media of the ancestor spirits that had passed away to communicate with the livings ( Sundermann, 1891).

The existence of *ere* ( *imam*) in the ceremony of *fondrakö* was not just symbolical or as the executor but also as the religion legitimation that confirmed that *fondrakö* was not just the product arranging customs, but it also covered spiritual cosmology involving the real world and the spirit world. The ancient Nias people believed that the world of the living was always overlapped with the world of the dead, inhabited by the spirit of ancestors. They believed that the supernatural power in the world was also related with the activities of the supernatural spirits. In the animism perspective, the spirits are believed to have power and always wish to communicate with those who are still alive through the mediation of an imam or a shaman ( Harrison-Buck & Freidel, 2021; Strijdom, 2021). The legitimation of *ere* in a ceremony confirms that all law provisions agreed upon on earth have the supernatural world consequences through the presence of ancestor spirits as witnesses, consent grantors, and also supervisors or punishers. MacGregor (2019) explained that community basically builds a system of belief through narrations explaining how the physical world was formed and how humans live in that world. The narrations are always accompanied with rituals and concepts that describe that humans do not live alone and require harmony with the supernatural world for their current and future lives. Based on this thinking, it is seen that the religious values of *fondrakö* are very strong because of its role in forming the system of belief of the community.

The main law contained in *fondrakö* is not far different from the provisions of the Book of Leviticus in the Moses’s Torah. The laws set forth contain the custom aspect (social rules) on one hand, and the Leviticus aspect (ritual and religious rules) on the other. Therefore, *fondrakö* can be seen as a doctrinal theological construction that is integrated into the social system. Referring to the explanation of Smart (1984, p. 9), doctrine is an effort to provide a system, explanation, and intellectual power toward the language of mythology and symbolism deriving from the belief and religious ritual. Relying on that opinion, *fondrakö* of Nias people is not only understood at the level of myths, symbolism, and rituals but also a doctrinal system at the practical level in the middle of the community ( Potapova, Danilova, Prasolov, Makarova, & Kryukova, 2018). This is the reason why fear and obedience toward the values of *fondrakö* survive in every generation of Nias people. Even though Christianity has taken over the belief system of the Nias, the doctrinal power of *fondrakö* in the social order of the community has never really disappeared. Up to this day, although the villages have changed their form, the relationship between them fuses in civilization, and although the village power shifts to modern government and the ethnic religion has been abandoned, the doctrinal values of *fondrakö* remain to the foundation in the social interaction and the way the community lives.

The content fragment of *fondrakö* Maenamolo, found at Gomo, the oldest of the megalithic area that became the first human community civilization witnessed in Nias, is one of the evidences parallel with the teachings of Christianity ( Hammerle, 2010). This content of *fondrakö* was read to public by *ere* as many as 27 times with a purpose, and the content could linger and was memorized by listeners. That fragment of *fondrakö* is as follows ( Gulo, 2011):

“A tiger, sir

On the land split stands fire as big as a sago tree

Becoming small, then disappearing

This is the seat of the child of *Lawalani Sanetua* (the determiner of all things)

This is the law, this is *Fondrakö*

1. Do not fornicate
2. Do not steal
3. Do not deviate
4. Do not kill the living beings
5. Do not take the property of your own people
6. The ancestor spirits from your father ( *lahu zanalawa ama*)

The ancestor spirits from your mother ( *lahu zanebua ono*)

Father, money existing in heavens is floating

Here is our real father

His substitution is here, a person called father,

He is the leader of the village, an imam, and a counselor”

When it is observed, the content of *fondrakö* is not far different from some of the law received by Moses for *Bani Isra’il* at the foot of Mount Sinai and known as The Ten Commandments ( Sailhamer, 2009). The first *fondrakö* is parallel with the seventh commandment that forbids fornicating, the second *fondrakö* is parallel with the eighth commandment that forbids the act of stealing, the fourth *fondrakö* is parallel with the sixth commandment that forbids murder, the fifth *fondrakö* is parallel with the tenth commandment that forbids the desire to keep the public property, and the sixth *fondrakö* is parallel with the obligation to respect parents, which is the content of the fifth commandment. That is why *fondrakö* reflects moral formation ( Sahidah, 2020) among those who accept and implement the law.

The hybridization concept is explained very well by Hedges (2021). According to Hedges (2021), the lived religion always has the chance to experience the hybrid process. The main emphasis does not lie in the belief system and religious texts but focuses on the activities and behavior that are more real, where the moral values are seen and practiced together ( Ganzevoort & Roeland, 2014). This is the reason behind the description of Hedges on the existence of a very significant relationship between transcendent matters (texts and the system of belief) and the reality (practice and behavior). According to this concept, a very good relationship can last between an institution and an individual to make it happen ( Hedges, 2021), which in this case is *fondrakö* as an institution and community as the gathering of individuals. Based on that thinking, it can be concluded that *fondrakö* as the highest consensus institution of Nias community is not an elite hegemonial institution or an institution separated from its supporting community. On the contrary, the concept of *fondrakö* introduces how an institution is formed from the wish of the community themselves in realizing sacrality and the relations that must be maintained both personally and related to worldly matters ( Ritzer & Stepnisky, 2019b). In this case, *fondrakö* can be positioned as a religious language in the social life order ( Alston, 2005). Therefore, drawing a firm line between social content and religious content in *fondrakö* cannot be done because they are mutually integrated. Both are united in the text and in the spirit forming them.

### A blessing or a curse

*Fondrakö* is ritual-constitutional. In its legitimation, a colossal custom party ( *owasa*) was held, and the feast was made for all the inhabitants of the village. This is the characteristic of *fondrakö* as the highest constitutional institution of the community ( Beatty, 2013). The peak of *owasa* is a stipulation. First are the stipulation and enforcement of law for the next generation ( *fondrakö fanotou*). This is done because the laws of *fondrakö* are binding and generational. *Fondrakö fanotou* also functions as the effort to preserve law for their children and grandchildren as the next generation. Second is the stipulation of the revised law ( *fondrakö fawuluni*). This law is stipulated with the awareness that the community, challenges, and needs experience development. In a different time and a new generation, law adjustment is required by making null and void the previous laws that are no longer relevant and new laws are formulated so that they become adaptive with the demand of the era ( Zebua, 1996).

As the constitutional institution, *fondrakö* has a legal aspect (which has to be complied with) and punishment (the consequence due to violation). Its position is equal with the senate in a political institution that holds the power and highest sovereignty in the community behavior control ( Bond, 2015) and the people democracy ( Kosmin, 2015). All legal products that have been stipulated in *fondrakö* are binding in the macro and micro level, and demand high compliance. Even though the laws are not present in writing, its complete content is considered as a convention. Although realizing the very grave legal consequences due to violation, the community fight to maintain the convention and guard it as a cultural heritage. This occurs because the customary law has been integrated into the social structure so that it can survive as the power of values since the birth of someone until the person’s demise ( Zaluchu, 2020). Hedges (2021, p. 25) defines this pattern characteristic as a ‘ *sui generis’* deriving from a Latin term meaning *of its own kind.* As an independent law, its existence becomes very strong, personal, and indispensable. Stipulation is as the final provision where none can refute, change, or reduce it. *Fondrakö* becomes the highest law that does not depend on or is influenced by other things, except by the group of the community where the law itself has been established.

The main philosophy behind the implementation of *fondrakö* is as a blessing and a curse. The dimension of blessing and curse can be seen from its name. *Fondrakö* comes from the word *rakö* meaning ‘stipulated through oath and bound with a curse’ ( Mendrofa, 1981). Zebua explained that that philosophy becomes one of the effective powers based on the compliance of the community. For those who implement and obey it, they will obtain a blessing passing down to their children and grandchildren. On the contrary, those who violate it will receive social sanction, and they and their descendants will be mocked and cursed in their entire life ( Zebua, 1996).

The stipulation of this *fondrakö* law resembles the ways of agreement stipulation occurring in the ancient Middle East area, where the parties agreeing stipulated an agreement by sacrificing an animal cut in certain ways ( Beaumont, 2012). The animal was cut equally, and the left part was separated from the right part. The parties entering into an agreement passed the animal body by continuing saying a blessing and a curse. It is a blessing when they obey the agreement, and it is a curse like the sacrificed animal being cut half if they violate the agreement. This rite was done with full awareness toward the obligation to maintain the law and its consequences. A similar thing happens with the stipulation of *fondrakö.* All village inhabitants gathered together outside the meeting hall ( *ahe gosali*). In the Middle East, the agreement stipulation only involves the parties in the agreement, but *fondrakö* involves more mass, takes place communally, and is under the supervision of the customary elders and an *imam.* The covenant was conducted together by the community (adults and children) that inhabit one village. The peak of the ceremony is the stipulation with the blessing-curse symbolism. In that ceremony, several sticks, a cock, and liquid tin were provided. The ceremony was led by *ere,* where the sticks were broken in front of public. Then the chicken was sacrificed with an unusual way. Its legs and wings were broken and was killed by twisting its neck. The hot tin liquid was then poured inside the animal. The *ere* then shouted loudly and declared to public, “whoever violates whatever has been stipulated in this Mondrakö, they will soon die (broken like the sticks), or tortured like the chicken whose legs and wings are broken and everything eaten by them will feel hot like the hot tin poured into the mouth of the chicken.” The sentence is also equipped with the curse “ *Lö mowa’a ba danö ba lö molehe ba mbanua*” meaning that they (who violate) will never have their offspring ( Gea, 2013).

Creating collective compliance with the approach of ‘blessing and curse’ is the characteristic of the society that builds its community with myths. Tylor (2010) called this as the characteristic of primitive culture that relies on animism and has the existential function for humans ( Dhavamony, 2016). In such a community, the members build social participation with magical elements so that interpretation is created. Therefore, myths are required as the active power in the community life, but they are also a reality lived by in the daily life ( Malinowski, 1948) and a part of primitive esthetics. In some certain cultures, and it is equipped with dances with magic nuance ( Weir, 2013). Actually, there is no logical relationship between *fondrakö* law violation and the symbolism of broken sticks or the sacrificed animal, even including also the consequence aspect to the offspring. This can be explained from the thinking of Eliade (2002) on ancient community. Rites and rituals are not just a procedure but also inclusive with the effort to provide in-depth meaning for the sake of accomplishing the purposes of the rites and rituals. The words a curse and a blessing are considered as the effort to materialize supernatural things believed in the cosmology and can sublimize in the real world ( Dufour & Boutaud, 2013; Eliade, 2002). Therefore, everyone bound in the covenant will build awareness to completely comply with the law on one hand, and on the other, maintain fear as a way to avoid a curse due to violation.

### Social binding and modernity challenges

The survival of the ancient law of *fondrakö* in the middle of Nias community is caused by its function in the social binding formation. Although it is a legal rule and a socio-religious law, the community treat *fondrakö* as the glue for social life. Zebua (1996, p. 46) clarified the *fondrakö* law in two parts. The first part is the provisions that govern customs. In this first part, everything on a human life cycle that starts from birth to the demise of the humans is arranged in the law and customary provisions - including arrangements regarding honor and social stratification in the community. Meanwhile, the second part of *fondrakö* is the arrangement on the law covering the marriage customary law, the law governing the economic life, the rules on crimes, criminal and civil laws, and the law on civics (arranging the governance and decision making in the power structure in a village). The classification shows that *fondrakö* plays a role in forming social structure, making every member of the community bound in a contract. As in a contract, its success is determined by the awareness of the community members to mutually bind each other and mutually have solidarity toward each other. The thinking of Durkheim on community formation in the social eye perspective ( Durkheim, 2018, p. 130; Ritzer & Stepnisky, 2019a, pp. 92–95; Strenski, 2015, p. 129) can be used to understand how *fondrakö* is seen as norms, faith, and values functioning to form community collective awareness. The main objective of this awareness is as the cohesion and social integration. Every individual is demanded to be an agent to create wholeness, unity, and order of the community to comply with the law. For Durkheim (1965), the formation of collective awareness only happens if every individual acts as an agent for the collective interest. The results are social and economic resistance, stability, and security of the group.

The research of Puccioni (2016) discovered a fact that ancient Nias community lived in the community groups called *öri.* Every *öri* was separated from each other and had a legal system, customs, and the autonomous government under the leadership of an elder named *tuhenöri.* The bilateral relationship among *öri* was basically bad. The competition in economy, customs, and area influence could lead to the act of conquering each other, ethnic war, and open hostility. If an *öri* failed to build social binding in its community, that village would be vulnerable toward internal disunity and would be easily conquered by other enemies of *öri.* Therefore, every *tuhenöri* was obliged to guard the wholeness of each *öri* using *fondrakö.* With it, dialectics occurred between collective awareness and individual awareness in producing social binding leading to the formation of social integration in an *öri.* The problem is that villages are no longer öri in the modern era and the government in a community has been taken over by a different elite. Will *fondrakö* become only a cultural heritage and has it been replaced with the values of modernity?

Nielsen (2003) that relied on the thinking of Durkheim (1995) and Martins & Guerra (2013) formulated an argument to answer those questions. The past community rites could experience a number of form changes in the modern religious practice through a rite proliferation. A number of shifts and adjustments happened without changing the meaning. Demerath (2003) specifically defined the shifts and adjustments as the sacrality process due to the occurrence of religious myth transformation to become a habit in a cultural commonplace. Simultaneously, the number of rites and the rituals of early forms can just change anytime. Their emotional intensity decreases due to social contour changes and other factors from the era because of complex social differentiation. Hence, it can be concluded that in the modern community, both in the religious scope and in the culture, proliferation is a natural thing happening as a way of the community to maintain the self-existence and group identity ( Buzan & Albert, 2010). Although at the beginning the rites only involved certain groups and did not play a role in the community widely ( Hirschle, 2012), the acceptance in the context of cultural commonplace kept taking place. Adaptation and modification happened in the form of the ritual and philosophical values integrated into the social order involving family, the state, workplaces, markets, and ethnic groups.

The Nias’s *fondrakö* experienced a similar process. The modern Nias community life can never be separated from the *fondrakö* philosophical values. In every social, cultural, and religious practices, specifically seen in the social decision making, community interest, consensus and agreement, life cycle initiation (birth, adult initiation, marriage, death), warning implementation, kinship relationship and social action, and moreover the religiosity aspect, the values of *fondrakö* are still maintained and practiced. This proves that *fondrakö* has changed from the rites and rituals of ethnic religion to become life philosophy that keeps living in every generation. Even though the face of the world has been colored by digitalization and the internet, the signs of contemporary progress do not reduce or get rid of the local wisdom of the modern Nias community. In the marriage initiation, for example, *ere* as the ethnic *imam* no longer plays a role and is replaced with a priest. However, the procession essence and the meaning of marriage are still maintained according to the *fondrakö’s* strict customary provisions ( Saputra, 2018; Zaluchu, 2020).

## Conclusion

It can be concluded that *fondrakö* for the modern Nias community is not merely a rite or a primitive cultural product. Traditional practice has experienced transformation to become point of view of life and a philosophical product integrated into the way to behave, social structure, and community spirituality. This is why the modern Nias people cannot be separated from their local wisdom values. This also refutes the general point of view that the world change into the digital culture that is free of values has shifted the existence of traditional values. What has happened in Nias becomes an example of how the local wisdom is able to survive and experience adaptation because of the decisions of the community themselves. The contemporary Nias community are capable of drawing a line of when to interact with the modern lifestyle that is all digitalized and when they must hold the *fondrakö’s* philosophical principles as the foundation of the way of life. Hybridization requires thinking as an adaptive solution in accommodating the local wisdom into modernity without making them contradictory to each other. Through the philosophy of *fondrakö* keeps on living in the contemporary Nias community, it can be seen that modern humans will tend to have orientation toward primitive forms both in the social context and in religion to maintain values and morality.

## Data Availability

All data underlying the results are available as part of the article and no additional source data are required.
