# A *Fondrakö* text: Cultural religious tradition and social integration of community

**DOI:** 10.12688/f1000research.127772.2

**Published:** 2024-07-25

**Authors:** Sonny E. Zaluchu

**Affiliations:** 1 Sosiologi Agama, Universitas Kristen Satya Wacana, Salatiga, Jawa Tengah, 50711, Indonesia; 2 Teologi, Sekolah Tinggi Teologi Baptis Indonesia, Semarang, Jawa Tengah, 50141, Indonesia

**Keywords:** local wisdom, fondrakö, sociology, social binding, Durkheim, community

## Abstract

Fondrakö is the local wisdom of the Nias ethnic community and has two functions. First are the oral religious holy book teaching ancestors' values and philosophy. Second is the highest consensus institution of the traditional society formulating and making decisions on the socio-religious law to guarantee the order of the community. This paper explains that social differentiation has happened in the modern era; philosophical values of *fondrakö* remain to live up to this day and experience the proliferation of rites. The argumentation built through this paper is that primitive culture will not simply disappear in the modern community just because it experiences accommodation and the decrease of emotional intensity into new forms. Social laws of primitive religion are not always contradictory and can run parallel with civilization.

## Introduction

Initially, the ethnic-religious texts did not have the early written form. The contemporary final form transforms from the traditional format of oral products or oral law collection. The research results of Tsuria, Bellar, Campbell, and Cho (2021) conclude that along with time, holy books and religious texts experienced a shift from oral composition uttered in religious meetings and rites to written texts in the form of copies on animal skin or papyrus paper and last to be written in a book format after the revolutionary invention of a printing device. In other words, ancient religious texts were built in the oral tradition ( Clark, 2005) and then developed into written documents and advanced printing technology ( Long & Long, 2017). Although text transmission has changed, Widder (2017, p. 2) emphasized that the power of those texts relies on the linguistic aspect more than its form. According to Widder (2017), language becomes the best medium for delivering religious messages by listening to what is talked about or delivered orally. Therefore, in various traditions, the transmission of spiritual messages and teachings has started in the oral method. Especially before the writing and printing technology took over, oral tradition was deeply rooted in how the community built its spirituality. Through language, communities define their belief system and create symbols to communicate in the system horizontally.

*Fondrakö* is one of the oral traditions developed in the ancient Nias ethnic community but is still influential in contemporary community life. The type, shape, and content vary in each village. However, its existence is intended as a sign and a tool for teaching life. The values and teachings in this tradition remain as local wisdom in the social life order of the Nias community, even though most Nias people have converted into Christian followers and have abandoned their ethnic religion ( Harefa, 2013; Mendrofa, 1981). The reality is the laws of *fondrakö* are in line with the teachings of Christianity and are even feared and obeyed more than religious instructions. This is a crucial matter because the values of *fondrakö* rely on ethnic religion and customs, not on the teachings of the Christian religion sourced from Divine revelations. *Fondrakö* is the cultural product of a primitive community, which at the beginning took root in myths, traditions, rituals, and natural power, and is pagan from the animism-dynamism perspective. Because the community tends to give more attention to traditional values than to the teachings of the holy book, clashes of values and behavior often happen, and there will be a combination of teachings or syncretism appears on the other. This occurs because the perspective of the local inhabitants, *fondrakö*, is not merely the value of arranging social relationships and kinship but also includes spiritual teachings from an oral ‘religious text.’ The relationship of both is inseparable. Since the Nias people live and actualize as a community, fondrakö becomes the referent value alongside the spiritual aspect.

This paper aims to comprehend why *fondrakö* has become a discourse power that keeps surviving in the modern era. Exploration of the values and power of fondrakö is expected to contribute to the anthropological studies of culture and religious sociology, which will complete the literature on the unification of culture and religion. For that, the first thing to do is to introduce the origin of *fondrakö* using two approaches: myths and sociology. Then, the discussion will dig out the power of fondrakö in its traditional roots as it became a covenant, social binding, and integration into a belief system or ethnic, religious spirituality. Not many have conducted a specific exploration of the law of *fondrakö* and analyzed it in sociology. Several research results reported are more about the practical implementation of *fondrakö* in various fields of life and do not touch the socio-religious approach as discussed in this paper. The research of Harefa (2013), for instance, only descriptively explained the concept of *fondrakö* as a customary product. The power of *fondrakö* is seen as a tradition producing local wisdom. Therefore, the research of Zega (2019) elaborated on the implementation of the cooperation workshop as arranged in fondrakö and modern leadership management. Markus (2004), although not explicitly reviewing *fondrakö*, in his thesis on intercultural missions related to arts and handicrafts, mentioned that *fondrakö* is associated with initiating a clan leader in Nias. Gulo (2011) conducted quite complete research, writing a thesis on the relationship between *fondrakö* and the concept of *böwö*, namely the customary bride price in the Nias wedding scheme. The research on *fondrakö* as social binding and spiritual construction is the gap that will be addressed in this paper. The temporary belief is that *fondrakö* is not only a customary entity but also part of a belief system that moves various strata of the community’s social life in a consensus.

## Methods

### Ethical considerations

This article followed all ethical standards for research without direct contact with human or animal subjects.

This paper will be arranged in four steps to achieve the research objectives. First, theoretically, sacred texts and books should be explained in a cultural context, and then the relationship between fondrakö and Nias people’s religiosity should be described. A more specific discussion in the third part concerns the blessing and curse of *fondrakö* as a moral and supernatural force that builds people’s fear of not violating it. The last section is an analysis of how *fondrakö* plays a role in the formation of social binding and modernity challenges. Discussions rely on primary data from the literature on Nias in the form of books and research results published in journals. Web searches and library studies were conducted to collect these books. All of these sources have become references cited in the article.

Thus, this research is qualitative Marrying (2004), carried out with a literature study to construct a theory about *fondrakö.* There are still very few references to *fondrakö* and Nias culture, so all books and articles researched on Nias topics must be used. Several parts of that research provide data and notes about *fondrakö.* The research began with initial observations in a traditional Nias village. The researcher unilaterally decided on a conventional village as the object of observation and observed social behavior in joint decision-making based on *fondrakö* values. Observations were also focused on seeking answers as to whether *fondrakö* was still valid and to what extent it had influenced community decision-making. The observation results become a phenomenon analyzed and constructed using the sociology of religion approach. This approach was chosen because the observation group is a Christian community. However, they still practice *fondrakö* traditional values even though they are aware that the values of the two have mixed in the people’s perspective. Decisions taken within the group are universal and binding and affect the social dynamics of society. The sociology of religion approach was chosen because *fondrakö* directly impacts social interaction and is part of a belief system. As is known, sociology looks at religion, not from the point of view of human relations with God (theology). In contrast, sociology sees religion as about society and its impact on society. From this perspective, the sociology of religion will explain how religion, both primitive religion through *fondrakö* and mainstream religion, collaborate in shaping culture, behavior, and social interactions. *Fondrakö* is part of the culture of the inclusive Nias people, which is directly related to religious life.

## Results

### Theory of religious holy books

Every religion has its religious holy book. Through the religious holy book, the followers have doctrinal guidance on the principles of belief, values, and statements on what they believe in, the teachings that are felt in and considered holy, the wisdom to live well both individually and in community, and the guidance to approach or understand the transcendent God ( Wilson, 1991). The religious holy books are not far different from the texts born in the form of oral literary works, which are then written in a book. According to Madigan (2013), what makes them different is their attitude and treatment toward the text. Madigan (2013) stated that when a community of believers considers and admits a text has sacredness and dignity and can be accepted as a truth to strengthen faith and religious life, the text is categorized as a religious holy book. Suppose it has been accepted as holy writing. In that case, the influence of the text spreads, not only reaching the religious area but also covering the community’s political, social, and economic life. Furthermore, Madigan explained that the texts in a holy book are essential elements in a complex process of religious follower identity formation ( Madigan, 2013). Through the texts in a holy book, religious followers can hear God in the middle of the community ( Siddiqee, 2013). In other words, the religious holy book texts are, on the one hand, informative and, on the other, performative because they are related directly to the religious follower community identity formation. The relation of both forms the locus of belief in the middle of the community. Therefore, the religious holy books always correlate with how religious followers express their social life ( Ritzer & Stepnisky, 2019a, p. 111). Using the above perspective, it can be seen that the written forms of Fondrako that are present in the ancient Nias society originated from oral tradition, which eventually functioned as a social and religious ‘guidebook’ in people’s lives.

In the beginning, religious holy books did not appear in a contemporary form. The information inside them as the content of a sacred book took a more primitive form when first introduced in society, namely utterances and oral communication. Durkheim stated that analyzing the contemporary religious form only manages to be done when that religion is learned in its most primitive form ( McKinnon, 2014). In the religious holy book context, the thinking of Durkheim can be accepted because the truth is the religious sacred books we receive today are the transformation results from their primitive form sourced from animism, naturism, and totemism ( Durkheim, 2011). In that primitive form, the religious holy books did not have their current form. Religious holy books’ informative and performative form is stated in symbols, abstractions, rituals, and other oral forms. As time went by, the texts experienced shifts and form change. In the beginning, they were delivered in oral and verbal composition. They were written and stated in the form of reading, then transferred in various forms of manuscript copies, and finally arrived in a contemporary form in a book or digital application ( Tsuria *et al.*, 2021).

By taking the examples of the dichotomy of holy-forbidden and holy-unholy from the context of The Old Testament, Hundley explained that religious texts, in general, provide clues to the followers on sacrality ( Hundley, 2013). Sacred space formed from religious texts becomes direction and guidance in living a pious life and behaving religiously in a secular world. This is inseparable from how the community interprets religious texts and applies them in their life. According to Sanders, the hermeneutic pattern always relies on two points. The first reference is a text as a tradition that becomes the behavior pattern in a context and situation ( Sanders, 1987, p. 173). In this case, it is seen that religious and social behavior are also influenced by how far a group of communities interprets their religious texts. Regarding this, Shillington ( Shillington, 2002, p. 63) revealed that religious texts are from the unity of language, texts, and oral utterances that encourage intention inside the religious followers to behave according to the content of their holy book. Therefore, religious sacred books can be seen as a rule that internalizes and binds the religious followers to carry out their role in the social order. Berger & Luckmann (2018, p. 21) supported this conclusion through his theory on social construction. Religious holy book texts can become an individual knowledge potential that can be developed to form reality in the middle of the community. We can conclude from that statement that social behavior parallels the aspect of community religiosity. Both exist in the dialectical relationship. Social behavior reflects the interpretation and internalization of religious values through religious holy books.

### *Fondrakö* and religiosity

Validation of *fondrakö* was conducted in a rite led by *ere.* The ancient Nias belief had a religious imam ( Laiya, 1983, p. 24). The meaning of *ere* as the local religion *imam* was also introduced by the missionaries that came to Nias when they spread the Christian religion ( Hummel & Telaumbanua, 2007; Sundermann, 1905; Zaluchu, 2021). The original meaning of *ere* is an expert or specialist in a particular field. Therefore, the function of *ere* was often associated with religious rites and rituals, so the position was made to be specific in the field of religion.

The activity center of *ere* was a meeting house named *osali.* In *osali*, *ere* led the ceremonies and rituals in front of the inhabitants and explained and interpreted the laws of the custom and religion. *Osali* did not only become a public space but also functioned as a religious space. Western missionaries adopted this idea when they changed the function of *osali* to become the worship place for Christians. Therefore, *they had a vital role in the rite management for the needs of the customs* and religion of the community. Its position was equal to that of a ruler and was very respected. *Ere* was even considered a holy person because he had the supernatural ability, namely as a mediator, to enter the spirit world. The *imam* was believed to be the traffic media of the ancestor spirits that had passed away to communicate with the living ( Sundermann, 1891).

The existence of *ere* ( *imam*) in the ceremony of *fondrakö* was not just symbolic or as the executor but also as the religious legitimation that confirmed that *fondrakö* was not just the product arranging customs, but it also covered spiritual cosmology involving the natural world and the spirit world. The ancient Nias people believed that the world of the living always overlapped with the world of the dead, inhabited by the spirit of ancestors. They thought that the supernatural power in the world was also related to the supernatural spirits’ activities. From the animist perspective, the spirits are believed to have control and always wish to communicate with those still alive through the mediation of an imam or a shaman ( Harrison-Buck & Freidel, 2021; Strijdom, 2021). The legitimation of *ere* in a ceremony confirms that all law provisions agreed upon on earth have supernatural world consequences through the presence of ancestor spirits as witnesses, consent grantors, and also supervisors or punishers. MacGregor (2019) explained that a community builds a belief system through narrations describing how the physical world was formed and how humans live in that world. The narrations are always accompanied by rituals and concepts that describe that humans do not live alone and require harmony with the supernatural world for their current and future lives. Based on this thinking, it is seen that the religious values of *fondrakö* are extreme because of their role in forming the system of belief of the community.

The primary law contained in *fondrakö* is not far different from the provisions of the Book of Leviticus in Moses’s Torah. The laws include the custom aspect (social rules) and the Leviticus aspect (ritual and religious rules). Therefore, *fondrakö* can be seen as a doctrinal, theological construction integrated into the social system. Referring to the explanation by Smart (1984, p. 9), the doctrine is an effort to provide a system, explanation, and intellectual power toward the language of mythology and symbolism deriving from the belief and religious ritual. Relying on that opinion, *the fondrakö of the* Nias people is not only understood at the level of myths, symbolism, and traditions but also a doctrinal system at the practical level in the middle of the community ( Potapova, Danilova, Prasolov, Makarova, & Kryukova, 2018). This is why fear and obedience toward the values of *fondrakö* survive in every generation of Nias people. Even though Christianity has taken over the belief system of the Nias, the doctrinal power of *fondrakö* in the community’s social order has never really disappeared. Even though the villages have changed their form, the relationship between them fuses in civilization. Although the village power has shifted to modern government and the ethnic religion has been abandoned, the doctrinal values of fondrakö remain the foundation of social interaction and the way the community lives. That is why the church in Nias is challenged to replace the values of violence, fear, and judgment inherited by the fondrako with teachings derived from the scriptures, such as love, forgiveness, repentance, and new life in Christ.

The content fragment of *fondrakö* Maenamolo, found at Gomo, the oldest of the megalithic area that became the first human community civilization witnessed in Nias, is one of the evidences parallel with the teachings of Christianity ( Hammerle, 2010). This content of *fondrakö* was read to the public by *ere* as many as 27 times with a purpose, and the content could linger and was memorized by listeners. That fragment of *fondrakö* is as follows ( Gulo, 2011):

“A tiger, sir

On the land split stands fire as big as a sago tree

Becoming small, then disappearing

This is the seat of the child of *Lawalani Sanetua* (the determiner of all things)

This is the law; this is *Fondrakö*

1. Do not fornicate
2. Do not steal
3. Do not deviate
4. Do not kill the living beings
5. Do not take the property of your people
6. The ancestor spirits from your father ( *lahu zanalawa ama*)

The ancestor spirits from your mother ( *lahu zanebua ono*)

Father, money existing in heavens is floating

Here is our real father

His substitution is here, a person called father,

He is the leader of the village, an imam, and a counselor.”

When observed, the content of fondrakö is not far different from some of the laws received by Moses for Bani Isra’il at the foot of Mount Sinai, known as The Ten Commandments ( Sailhamer, 2009). The first *fondrakö* is parallel with the seventh commandment that forbids fornicating; the second *fondrakö* is parallel with the eighth commandment that prohibits the act of stealing; the fourth *fondrakö* is parallel with the sixth commandment that forbids murder; the fifth *fondrakö* is parallel with the tenth commandment that forbids the desire to keep the public property, and the sixth *fondrakö* is parallel with the obligation to respect parents, which is the content of the fifth commandment. That is why *fondrakö* reflects moral formation ( Sahidah, 2020) among those who accept and implement the law.

Hedges (2021) explains the hybridization concept very well. According to Hedges (2021), the lived religion always has the chance to experience the hybrid process. The main emphasis does not lie in the belief system and religious texts. Still, it focuses on the activities and behaviors that are more real, where the moral values are seen and practiced together ( Ganzevoort & Roeland, 2014). This is the reason behind Hedges’s description of a significant relationship between transcendent matters (texts and the system of belief) and reality (practice and behavior). According to this concept, a perfect relationship can last between an institution and an individual to make it happen ( Hedges, 2021), which in this case is *fondrakö* as an institution and community as the gathering of individuals. Based on that thinking, it can be concluded that *fondrakö*, as the highest consensus institution of the Nias community, is not an elite hegemonial institution or an institution separated from its supporting community. On the contrary, the concept of *fondrakö* introduces how an institution is formed from the community’s wish to realize sacrality and the relations that must be maintained personally and related to worldly matters ( Ritzer & Stepnisky, 2019b). In this case, *fondrakö* can be positioned as a religious language in the social life order ( Alston, 2005). Therefore, drawing a firm line between social and spiritual content in *fondrakö* cannot be done because they are mutually integrated. Both are united in the text and in the spirit forming them.

### A blessing or a curse

*Fondrakö* is ritual-constitutional. In its legitimation, a colossal custom party ( *owasa*) was held, and the feast was made for all the village inhabitants. This is the characteristic of *fondrakö* as the highest constitutional institution of the community ( Beatty, 2013). The peak of *owasa* is a stipulation. First are the stipulation and enforcement of the law for the next generation ( *fondrakö fanotou*). This is done because the laws of *fondrakö* are binding and generational. *Fondrakö fanotou* also functions as the effort to preserve law for their children and grandchildren as the next generation. Second is the stipulation of the revised law ( *fondrakö fawuluni*). This law is stipulated with the awareness that the community, challenges, and needs experience development. In a different time and with a new generation, law adjustment is required by making null and voiding the previous laws that are no longer relevant. New laws are formulated to become adaptive to the era’s demands ( Zebua, 1996).

As a constitutional institution, *fondrakö* has a legal aspect (which has to be complied with) and punishment (the consequence due to violation). Its position is equal to that of the Senate, a political institution with the power and highest sovereignty in community behavior control ( Bond, 2015) and the people’s democracy ( Kosmin, 2015). All legal products stipulated in *fondrakö* are binding at the macro and micro level and demand high compliance. Even though the laws are not present in writing, their complete content is considered a convention. Although realizing the grave legal consequences due to violation, the community fights to maintain the convention and guard it as a cultural heritage. This occurs because customary law has been integrated into the social structure to survive as the power of values from the birth of someone until the person’s demise ( Zaluchu, 2020). Hedges (2021, p. 25) defines this pattern characteristic as a ‘ *sui generis’* deriving from a Latin term meaning *of its kind.* As an independent law, its existence becomes vital, personal, and indispensable. The stipulation is the final provision that no one can refute, change, or reduce. *Fondrakö* becomes the highest law that does not depend on or is influenced by other things except by the community group where the law itself has been established.

The main philosophy behind implementing *fondrakö* is that it is a blessing and a curse. The dimension of blessing and curse can be seen from its name. *Fondrakö* comes from the word *rakö*, which means ‘stipulated through oath and bound with a curse’ ( Mendrofa, 1981). Zebua explained that philosophy has become one of the influential powers based on community compliance. Those who implement and obey it will obtain a blessing passed down to their children and grandchildren. On the contrary, those who violate it will receive social sanction, and their descendants will be mocked and cursed their entire lives ( Zebua, 1996).

The stipulation of this *fondrakö* law resembles the ways of agreement stipulation occurring in the ancient Middle East area, where the parties stipulated an agreement by sacrificing an animal cut in specific ways ( Beaumont, 2012). The animal was cut equally, and the left was separated from the right. The parties agreeing passed the animal body by continuing to say a blessing and a curse. It is a blessing when they obey the agreement, and it is a curse, like the sacrificed animal being cut in half if they violate the deal. This rite was done with full awareness of the obligation to maintain the law and its consequences. A similar thing happens with the stipulation of *fondrakö.* All village inhabitants gathered together outside the meeting hall ( *the goal*). In the Middle East, the agreement stipulation only involves the parties. Still, fondrakö involves more mass, takes place communally, and is supervised by the customary elders and an *imam.* The covenant was conducted together by the community (adults and children) that inhabit one village. The peak of the ceremony is the stipulation with the blessing-curse symbolism. In that ceremony, several sticks, a cock, and liquid tin were provided. The ceremony was led by *ere,* where the sticks were broken in front of the public. Then, the chicken was sacrificed unusually. Its legs and wings were broken, and it was killed by twisting its neck. The hot tin liquid was then poured into the animal. The *ere* then shouted and declared to the public, “Whoever violates whatever has been stipulated in this Mondrakö, they will soon die (broken like the sticks), or tortured like the chicken whose legs and wings are broken and everything eaten by them will feel hot like the hot tin poured into the mouth of the chicken.” The sentence is also equipped with the curse “ *Lö mowa’a ba danö ba lö molehe ba mbanua,*” meaning that they (who violate) will never have their offspring ( Gea, 2013).

Creating collective compliance with the approach of ‘blessing and curse’ is the characteristic of the society that builds its community with myths. Tylor (2010) called this the characteristic of primitive culture that relies on animism and has an existential function for humans ( Dhavamony, 2016). In such a community, the members build social participation with magical elements so that interpretation is created. Therefore, myths are required as active power in community life, but they are also a reality lived by daily life ( Malinowski, 1948) and a part of primitive esthetics. In certain cultures, it is equipped with dances with magic nuance ( Weir, 2013). There is no logical relationship between *fondrakö* law violation and the symbolism of broken sticks or the sacrificed animal, even including the consequence to the offspring. This can be explained by Eliade’s (2002) thinking about ancient communities. Rites and rituals are not just a procedure but also inclusive with the effort to provide in-depth meaning to accomplish the purposes of the rites and rituals. The words a curse and a blessing are considered as the effort to materialize supernatural things believed in cosmology and can sublimize in the real world ( Dufour & Boutaud, 2013; Eliade, 2002). Therefore, everyone bound in the covenant will build awareness to comply with the law entirely and, on the other hand, maintain fear to avoid a curse due to violation.

### Social binding and modernity challenges

The survival of the ancient law of *fondrakö* in the middle of the Nias community is caused by its function in the social binding formation. Although it is a legal rule and a socio-religious law, the community treats *fondrakö* as the glue for social life. Zebua (1996, p. 46) clarified the *fondrakö* law in two parts. The first part is the provisions that govern customs. In this first part, everything in a human life cycle from birth to the demise of humans is arranged in the law and customary provisions, including arrangements regarding honor and social stratification in the community. Meanwhile, the second part of *fondrakö* is the arrangement of the law covering marriage customary law, the law governing economic life, the rules on crimes, criminal and civil laws, and the law on civics (arranging the governance and decision-making in the power structure in a village). The classification shows that *fondrakö* plays a role in forming social structure, making every community member bound by a contract. As in a contract, its success is determined by the community members’ awareness of binding each other and having solidarity toward each other. The thinking of Durkheim on community formation from the social eye perspective ( Durkheim, 2018, p. 130; Ritzer & Stepnisky, 2019a, pp. 92–95; Strenski, 2015, p. 129) can be used to understand how *fondrakö* is seen as norms, faith, and values functioning to form community collective awareness. The main objective of this awareness is cohesion and social integration. Every individual is demanded to be an agent to create wholeness, unity, and order in the community to comply with the law. For Durkheim (1965), the formation of collective awareness only happens if every individual acts as an agent for the collective interest. The results are social and economic resistance, stability, and security of the group.

Puccioni (2016) discovered that the ancient Nias community lived in a group called *öri.* Every *öri* was separated from each other and had a legal system, customs, and an autonomous government under the leadership of an elder named *tuhenöri.* The bilateral relationship among *öri* was terrible. The competition in economy, customs, and area influence could lead to the act of conquering each other, ethnic war, and open hostility. If an *öri* failed to build social binding in its community, that village would be vulnerable to internal disunity and easily conquered by other enemies of *öri.* Therefore, every *tuhenöri* was obliged to guard the wholeness of each *öri* using *fondrakö.* With it, dialectics occurred between collective and individual awareness in producing social binding, leading to social integration in an *öri.* The problem is that villages are no longer öri in the modern era, and a different elite has taken over the government of a community. Will *fondrakö* become only a cultural heritage, and has it been replaced with modern values?

Nielsen (2003), who relied on the thinking of Durkheim (1995) and Martins & Guerra (2013), formulated an argument to answer those questions. The past community rites could experience several changes in modern religious practice through rite proliferation. Several shifts and adjustments happened without changing the meaning. Demerath (2003) specifically defined the shifts and adjustments as the sacrality process due to the occurrence of religious myth transformation to become a habit in a cultural commonplace. Simultaneously, the number of rites and early-form rituals can change over time. Their emotional intensity decreases due to social contour changes and other factors from the era because of complex social differentiation. Hence, it can be concluded that in the modern community, both in the religious scope and in the culture, proliferation is a natural thing happening as a way for the community to maintain self-existence and group identity ( Buzan & Albert, 2010). Although the rites only involved certain groups at the beginning and did not play a wide role in the community ( Hirschle, 2012) acceptance in the context of cultural commonplace continued. Adaptation and modification took the form of ritual and philosophical values integrated into the social order involving the family, the state, workplaces, markets, and ethnic groups.

The Nias’s *fondrakö* experienced a similar process. The modern Nias community life can never be separated from the *fondrakö* philosophical values. In every social, cultural, and religious practice, seen explicitly in the social decision-making, community interest, consensus and agreement, life cycle initiation (birth, adult initiation, marriage, death), warning implementation, kinship relationship, and social action, and the religiosity aspect, the values of *fondrakö* are still maintained and practiced. This proves that *fondrakö* has changed from the rites and rituals of ethnic religion to a life philosophy that keeps living in every generation. Even though the face of the world has been colored by digitalization and the internet, the signs of contemporary progress do not reduce or eliminate the local wisdom of the modern Nias community. In the marriage initiation, for example, *ere* as the ethnic *imam* no longer plays a role and is replaced with a priest. However, the essence of the process and the meaning of marriage are still maintained according to the *fondrakö’s* strict customary provisions ( Saputra, 2018; Zaluchu, 2020). However, modern Nias people no longer live in the past. Universal human values, the presence of law, and Christian teachings are also variables that shape the way of life of the young generation of Nias. The change does not marginalize fondrako values but undergoes a kind of natural selection in the way of thinking of the community, which of the local wisdom is still relevant and which is not.

## Conclusion

It can be concluded that *fondrakö* for the modern Nias community is not merely a rite or a primitive cultural product. Traditional practice has experienced transformation to become a point of view of life and a philosophical product integrated into the way to behave social structure, and community spirituality. This is why the modern Nias people cannot be separated from their local wisdom values. This also refutes the general point that the world’s change into a digital culture that is free of values has shifted the existence of traditional values. What happened in Nias becomes an example of how local wisdom can survive and experience adaptation because of the community’s decisions. The contemporary Nias community is capable of drawing a line between when to interact with the modern lifestyle that is all digitalized and when they must hold the *fondrakö’s* philosophical principles as the foundation of the way of life. Hybridization requires thinking as an adaptive solution to accommodate local wisdom into modernity without contradicting it. Through the philosophy of *fondrakö*, which keeps on living in the contemporary Nias community, it can be seen that modern humans tend to have an orientation toward primitive forms both in the social context and in religion to maintain values and morality.

## Data availability

All data underlying the results are available in the article; no additional source data are required.
