# Supplementary material for: A Fondrakö text: Cultural religious tradition and social integration of community
Source: F1000Res. 2024 Sep 17;12:41. Originally published 2023 Jan 11. [Version 3] doi: 10.12688/f1000research.127772.3 (PMC11393527; doi:10.12688/f1000research.127772.3)
Supplement: Peer review history

# Reviewer report: Reviewer response for version 3

**DOI:** 10.5256/f1000research.171884.r324421

**Published:** 2024-10-09

**Authors:** Nadia Sigi Prameswari

**Affiliations:** 1 Universitas Negeri Semarang, Semarang, Central Java, Indonesia

"I feel this manuscript has been revised and aligns with the review comments I provided earlier. Especially, the methods section has been explained in more detail."

Is the work clearly and accurately presented and does it cite the current literature?

Partly

If applicable, is the statistical analysis and its interpretation appropriate?

Partly

Are all the source data underlying the results available to ensure full reproducibility?

Partly

Is the study design appropriate and is the work technically sound?

No

Are the conclusions drawn adequately supported by the results?

No

Are sufficient details of methods and analysis provided to allow replication by others?

No

Reviewer Expertise:

Arts and Humanities

I confirm that I have read this submission and believe that I have an appropriate level of expertise to confirm that it is of an acceptable scientific standard.

# Reviewer report: Reviewer response for version 2

**DOI:** 10.5256/f1000research.169445.r316568

**Published:** 2024-09-11

**Authors:** Nadia Sigi Prameswari

**Affiliations:** 1 Universitas Negeri Semarang, Semarang, Central Java, Indonesia

Please explain the research design in the abstract and methods sections. In the methods section, it is mentioned that the research was conducted through a literature review, but this is not sufficient. Qualitative research needs to specifically explain which approach was used.

 It is also mentioned that data collection for this research was done through observation, but it falls short of my expectations, which should be rich in observational data evidenced by narratives within a specific setting, descriptions of the atmosphere, and supported by photographic documentation.

Is the work clearly and accurately presented and does it cite the current literature?

Partly

If applicable, is the statistical analysis and its interpretation appropriate?

Partly

Are all the source data underlying the results available to ensure full reproducibility?

Partly

Is the study design appropriate and is the work technically sound?

No

Are the conclusions drawn adequately supported by the results?

No

Are sufficient details of methods and analysis provided to allow replication by others?

No

Reviewer Expertise:

Arts and Humanities

I confirm that I have read this submission and believe that I have an appropriate level of expertise to confirm that it is of an acceptable scientific standard, however I have significant reservations, as outlined above.

# Reviewer report: Reviewer response for version 1

**DOI:** 10.5256/f1000research.140313.r159966

**Published:** 2024-09-12

**Authors:** Fransiskus Irwan Widjaja

**Affiliations:** 1 Sekolah Tinggi Teologi REAL, Batam, Indonesia

The author is courageous to expose and lift the fondrakö as a central research topic. This primitive oral culture still exists today and is very important to dig up and valuable for others concerned with ethnography or contextualizing the local culture using ancient history to study further. Zaluchu ‘s conclusion of the Social laws of primitive religion is not always contradictory. It can run parallel with civilization, and primitive culture will not simply disappear in the modern community is an audacious statement that should get two thumbs for appreciation. The author may look at other articles for comparison and additional citations attached.

Is the work clearly and accurately presented and does it cite the current literature?

Yes

If applicable, is the statistical analysis and its interpretation appropriate?

I cannot comment. A qualified statistician is required.

Are all the source data underlying the results available to ensure full reproducibility?

Yes

Is the study design appropriate and is the work technically sound?

Yes

Are the conclusions drawn adequately supported by the results?

Yes

Are sufficient details of methods and analysis provided to allow replication by others?

Yes

Reviewer Expertise:

Missiology, Ethnography and Theology of Mission

I confirm that I have read this submission and believe that I have an appropriate level of expertise to confirm that it is of an acceptable scientific standard.

## References

[1] : Realities and challenges for mission transformation in Sabu people. *HTS Teologiese Studies / Theological Studies* .2022;78(4) : 10.4102/hts.v78i1.7137 10.4102/hts.v78i1.7137

[2] : Contextual Mission Construction according to the Jubata Concept in the Kanayatn Dayak Tribe in Introducing the Triune God. *International Journal of Indonesian Philosophy & Theology* .2022;3(2) : 10.47043/ijipth.v3i2.34 10.47043/ijipth.v3i2.34

# Reviewer report: Reviewer response for version 1

**DOI:** 10.5256/f1000research.140313.r185544

**Published:** 2023-07-28

**Authors:** Oinike Natalia Harefa

**Affiliations:** 1 STT BNKP Sundermann, Gunungsitoli, Indonesia

1. There are many kinds of fondrakö in Nias that are established based on the area (villages). It is better to make it clear from the beginning that each fondrakö is not for the whole Nias people.

 2. In the past, fondrakö delivered with oral tradition. Today, some fondrakö have been written. What do you think about this? I suggest that the author emphasize this relation in the section of the holy book.  

 3. Does decision-making through the fondrakö deal with justice for the victims of violence? Or is it more for the social interest rather than a preference for individual well-being? For instance, in the case of violence against women.

 4. The author mentions about "dowry" in Nias. Please check the right terminology for this, is it dowry or bride price? It is two different things. 

 5.  The author mentions how fondrakö affects the relationship with God, this tradition is more judgemental rather than a grace-based approach, as the soul of Christianity. Even, the author also writes, "This is the reason why fear and obedience toward the values of fondrakö survive in every generation of Nias people". How does the author deal with this?

 6.  Is the fondrakö still relevant today for the younger generation in Nias? The author seems too optimistic about fondrakö but less in criticism, especially at the point of blessing or curse.

Is the work clearly and accurately presented and does it cite the current literature?

Yes

If applicable, is the statistical analysis and its interpretation appropriate?

I cannot comment. A qualified statistician is required.

Are all the source data underlying the results available to ensure full reproducibility?

Partly

Is the study design appropriate and is the work technically sound?

Partly

Are the conclusions drawn adequately supported by the results?

Yes

Are sufficient details of methods and analysis provided to allow replication by others?

Yes

Reviewer Expertise:

Indonesia

I confirm that I have read this submission and believe that I have an appropriate level of expertise to confirm that it is of an acceptable scientific standard, however I have significant reservations, as outlined above.
